# Single-Particle Tracking with Scanning Non-Linear Microscopy

**DOI:** 10.3390/nano10081519

**Published:** 2020-08-03

**Authors:** Théo Travers, Vincent G. Colin, Matthieu Loumaigne, Régis Barillé, Denis Gindre

**Affiliations:** MOLTECH-Anjou Laboratory, UMR CNRS 6200, University of Angers, 2 bd Lavoisier, 49045 Angers CEDEX, France; theo.travers@univ-angers.fr (T.T.); vincent.colin@univ-angers.fr (V.G.C.); matthieu.loumaigne@univ-angers.fr (M.L.); regis.barille@univ-angers.fr (R.B.)

**Keywords:** non-linear microscopy, two-photon fluorescence imaging, scanning microscopy, single particle tracking, free diffusion, particle size characterization, particle tracking algorithms

## Abstract

This study describes the adaptation of non-linear microscopy for single-particle tracking (SPT), a method commonly used in biology with single-photon fluorescence. Imaging moving objects with non-linear microscopy raises difficulties due to the scanning process of the acquisitions. The interest of the study is based on the balance between all the experimental parameters (objective, resolution, frame rate) which need to be optimized to record long trajectories with the best accuracy and frame rate. To evaluate the performance of the setup for SPT, several basic estimation methods are used and adapted to the new detection process. The covariance-based estimator (CVE) seems to be the best way to evaluate the diffusion coefficient from trajectories using the specific factors of motion blur and localization error.

## 1. Introduction

Single-particle tracking (SPT) is a well-known method to measure the motion of sub-micrometer particles in a liquid phase. This technique provides access to information about particle size which cannot be reached by imaging because of the diffraction limit. Estimating the particle localization and performing statistical calculations over many frames makes it possible to estimate its displacements with high accuracy and to measure the diffusion coefficient [1]. This coefficient is linked directly to the size of the particles and allows estimation of a size depending on the movement recorded [2].

SPT method has proven its worth with fluorescence microscopy since T. Schmidt in 1996 [3] with the first imaging of a moving fluorescent particle. This experiment was achieved with wide-field imaging illuminated by a laser at 514 nm and a high signal-to-noise ratio localization ($SNR_{loc}$) of 28. With a localization accuracy of 30 nm, the experiment is a reference for SPT methods. Since then, many improvements have been made. Two different approaches have been developed: one involves studying the behavior of a single-particle over a long time (SPT) [4], the other one, called Nanoparticle Tracking Analysis, uses the statistical advantage of a large number of particles to estimate the size distribution [5]. The SPT is a useful method for determining the radius of free-scattering nanoparticles but also for dissecting different kind of motions of fluorescently labelled moieties, and this has led to the development of many applications in biology and biophysics in the past few years [6,7].

Thanks to new materials and methods developed since the first experiment, it is now possible to localize particles with an accuracy of a few nanometers (≈10 nm) [8]. New methods for the estimation of the diffusion coefficient, such as the covariance-based estimator [9], have been an important research topic to find the optimal experimental parameters and the best way to use the information about the movement of particles in a liquid phase.

These optimal parameters and methods have been tested and validated with various experiments and simulations [10,11,12], but always using single-photon fluorescence and wide-field microscopy.

The usual particle tracking techniques based on a sequence composed of many wide-field images cannot be applied to this type of microscopy. Indeed, Non-Linear Optical (NLO) responses of materials, like Two-Photon Excited Fluorescence (TPEF) or Second-Harmonic Generation (SHG), require high intensity excitation; direct imaging of the sample is therefore possible with temporal focusing method but requires cutting-edge equipment and setup [13,14]. SPT can be used from confocal images obtained with scanning microscopy [15]. For non-linear imaging, the commonly used method is to scan a small area with a pulsed laser, then the non-linear response of the sample is recorded and reconstructed to create a pixel-by-pixel image. The full process leading to the final image is lengthy and represents a limit to track moving particles. For these reasons, only a few experiments have been performed on the tracking of particles with non-linear microscopy. The article of Gregor [16] in 2017 is the first to introduce rapid non-linear image scanning microscopy as a method to track particles with a frame rate of 30 Hz. However, in this study, the tracking of particles is only performed with very short track (<10 positions). Two-photon microscopy was also used in 3D tracking techniques [17]. The purpose of this study is to track particles over a long period of time to estimate their sizes. This study shows that it is possible to obtain good size estimation with non-linear microscopy tracking on calibrated particles smaller than 1 μm by optimizing the scanning and optical parameters.

This nonintuitive approach inevitably has limitations compared to full-field imaging techniques: the image resolution has to be low in order to reduce the time between frames, the dwell time (i.e., time spent on a pixel by the laser) on each pixel must be sufficient to detect a few NLO photons, and the signal-to-noise ratio is lower [18] than single-photon microscopy. However, this approach has several advantages: not only does it provides access to NLO particle tracking, but it also reduces the phenomenon of photobleaching in the case of fluorescent particles, due to the smaller intrinsic focal volume compared with single-particle fluorescence. Moreover, the near-infrared wavelength used in two-photon microscopy provides a better depth penetration in tissues with less toxicity on living cells. These two advantages make two-photon imaging a more suitable technique for biology [19].

Another type of two-photon microscopy is the Second-Harmonic Imaging Microscopy [20] based on the first demonstration of SHG performed in 1961 [21]. For special materials which have non-centrosymmetric properties, the SHG process exactly doubles the wave frequency for high intensity excitation. This phenomenon is a direct conversion of energy without absorption which means there is no photobleaching effect during imaging.

It is relevant to note that other optical techniques for particle sizing using non-linear effects have also been developed, for instance non-linear correlation spectroscopy [22]. This technique, akin to fluorescence correlation spectroscopy, could be seen as SPT but with only one pixel. While this technique is arguably simpler to implement and allows study of smaller particles, it is only devoted to particle sizing and cannot recreate an image.

The SPT method has many applications in biology and particularly in cellular activity and virology. Using non-linear microscopy to track biomarker in living cells without the limitation of single-photon imaging could help to better understand biological mechanisms [23]. Non-linear microscopy is a powerful tool with high resolution if the typical moving time of the sample is far longer than the time required to create the image.

The goal here is to demonstrate the efficiency of the SPT method with non-linear microscopy. It has several advantages compared to single-photon microscopy but also many limits which call for compromises.

As a first step, the scanning signal was optimized to generate usable images for post-processing. To evaluate the relevance of this new application, three basic statistical methods are used here to estimate the size of particles. Future studies will focus on improving size estimation with innovative methods such as sigma point-based expectation maximization [24] or the maximum likelihood estimator [25].

## 2. Material and Methods

### 2.1. Equipment

The laser used is a Ti:Sa femtosecond pulsed laser operating between 700 and 1000 nm (Tsunami, Spectra-Physics, Santa Clara, CA, USA). To achieve the scanning process, a mirror galvanometer (6210H, Cambridge Technology, Bedford, MA, USA)) is used to control the angle of the laser beam. A scan lens (LSM54-850, Thorlabs Inc., Newton, NJ, USA) and tube lens are placed to (i) create an afocal optical system, (ii) enlarge the beam diameter for widening the illuminated part of the objective pupil aperture (in order to increase the resolution) and conjugates the mirrors and the objective pupil plane, (iii) create a conjugated plane between the primary image plan (in the focal plan of the scan lens) and the scanned area of the sample (See Figure S1 of Supplementary Materials).

The separation of excitation and emission signals is achieved by a longpass dichroic mirror (DMLP490R, Thorlabs Inc., Newton, NJ, USA); however a Bandpass filter (377/50 Bandpass Filter, Semrock Inc., New York, NY, USA) with high optical density is required to eliminate most of the residual signal of the excitation source. The nonlinear response of the sample is collected by a superfast wideband photomultiplier (H7422P-40, Hamamatsu, Japan).

Where static imaging is based on image accumulation, moving objects imaging requires raising the intensity of the laser source to increase the number of photons emitted by the sample. The high optical density filter should stop all the residual photons coming from the dichroic mirror but with a very intense excitation source, a few photons still reach the photomultiplier (PM), thus adding noise.

A microscope objective is used for both focusing the excitation beam towards the sample and collecting the signal generated by non-linear processes. The objective used is a X20, NA = 0.75 (UPLSAPO20X, Olympus Corp., Japan); the reasons for choosing this objective will be discussed later in Section 4.

**Figure 1:**
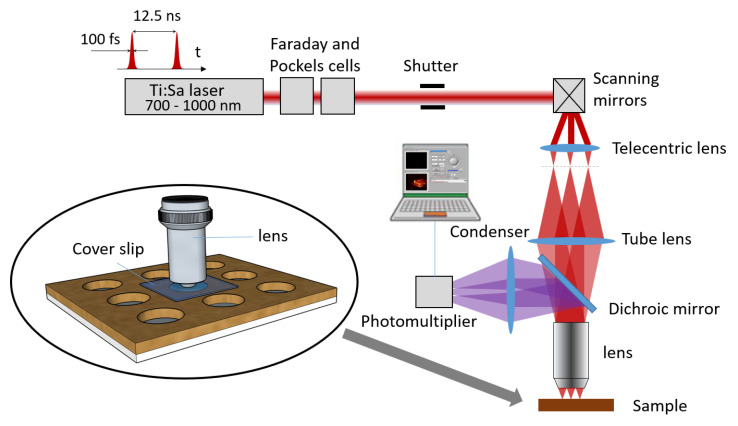
Complete setup of the non-linear microscope, which can be used for two-photon fluorescence and second-harmonic generation. The circled diagram shows the medium used to study the particles. The solvent containing the particles fills one of the cells of a multiwell support (Silicone Isolators™, GBL664206, Grace Bio-Labs) and a microscope slide is placed between the study volume and the microscope objective. Thus, turbulence due to evaporation of the liquid does not occur.

Figure 1 shows the complete setup of the scanning microscope. More information about the setup can be found in Section S1 of Supplementary Materials.

### 2.2. Consumables

To demonstrate the feasibility of this approach, we used calibrated commercial fluorescent particles. The fluorescent latex beads used for the experiments are Fluoresbrite${}^{®}$ (24054, PolyScience, Niles, IL, USA) of 250 nm and 110 nm radius, with absorption and emission maxima at 377 nm and 479 nm for both sizes, and a satisfying size dispersion of 3% and 5% respectively. To study these beads correctly, a few important steps are taken. First, a 1000-fold dilution ensures that each particle’s free diffusion is long enough to record tracks without collisions. Then the diluted solution is placed for 5 min in an ultrasonic bath to minimize the number of aggregated particles. Finally, a few droplets of the solution are poured in a small 4.5 mm diameter well formed by a silicone isolator stuck to a coverslip. A droplet bigger than the well must be formed to add an extra coverslip on the top and ensure that no evaporation will occur and disturb the observations.

### 2.3. Software

The whole setup is managed by an in-house engineered interface in LabVIEW (National Instruments, Austin, TX, USA), a highly modular graphical programming language for data acquisition and monitoring environments. The program controls the whole process from the positioning of the scanning mirror until the image acquisition. The sequence of images is then processed in a particle tracking plug-in of ImageJ [26] called TrackMate [27] to build all the individual trajectories and extract a data file. A simple Matlab program (The MathWorks Inc., Natick, MA, USA) is used to transform the successive positions into an estimation of the diffusion coefficient.

### 2.4. Scanning Methods

**Figure 2:**
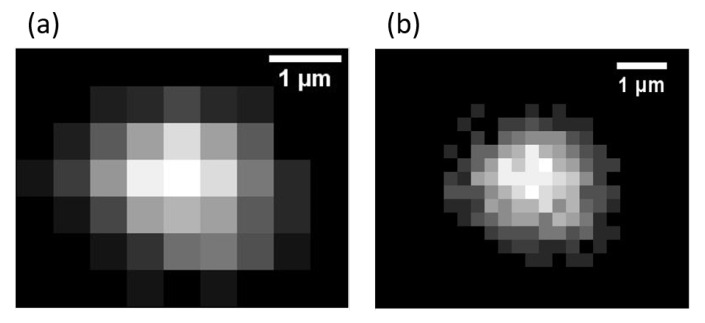
Images of the same bead (radius = 250 nm) with (**a**) 48,260 dpi (1.9 pixels/μm) resolution and (**b**) with 96,520 dpi (3.8 pixels/μm) resolution.

To create the image of an area, a non-linear microscope needs to light up several successive points and then rebuild the signal response detected by the photomultiplier. Each step of the scanning process creates one pixel of the image. The spatial resolution, limited by diffraction, does not allow nanoparticles to be resolved. But the image resolution (i.e., the total number of pixels in the image) is an essential factor of accuracy for the localization of each particle. According to the Nyquist sampling theorem, at least two pixels is needed to describe the radius of the Point Spread Function (PSF). If *p* is the pixel size and *s* the PSF radius, then the criteria $\pi {\left(2p\right)}^{2}\approx 12p^{2}\leq s^{2}$ must be respected. As shown in Figure 2 the image (a) of the particle is slightly under-sampled whereas image (b) respects the Nyquist sampling limit.

**Figure 3:**
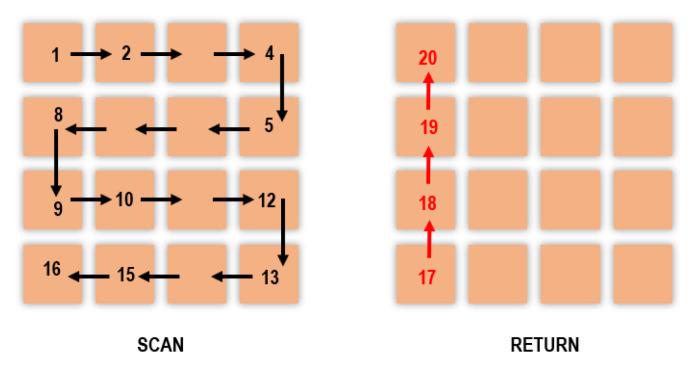
Drawing of the scanning process. Each orange square represents a residence time of the laser. During this dwell time, the photomultiplier counts the number of incoming photons, thus determining the pixel value of the final image. The laser beam follows the black arrows over the area to create an image. The red arrows show the path back to the starting position for a controlled dead time. In this simplistic example of a 4 × 4 pixel image, the return to the starting position requires 4 additional dwell times.

Forming an image from several successive spots takes some time. Therefore, the scanning method must be optimized to reduce the image acquisition time. Several scanning methods were tested in order to reduce the dead times, i.e., the times when the mirrors return to the beginning of the line or to the starting point without being able to detect a useful signal. A discussion of the different methods is detailed in Section S2 of Supplementary Materials. In order to optimize the duration of a sequence and to keep the same time between two images, the chosen solution, illustrated in Figure 3, requires the laser to scan line by line (once on the last position of one row, the laser starts scanning the following line in the reverse direction). Coming back to the starting point is achieved through a controlled dead time by scanning linearly the first column without recording the signal.

### 2.5. Track Analysis Methods

**Figure 4:**
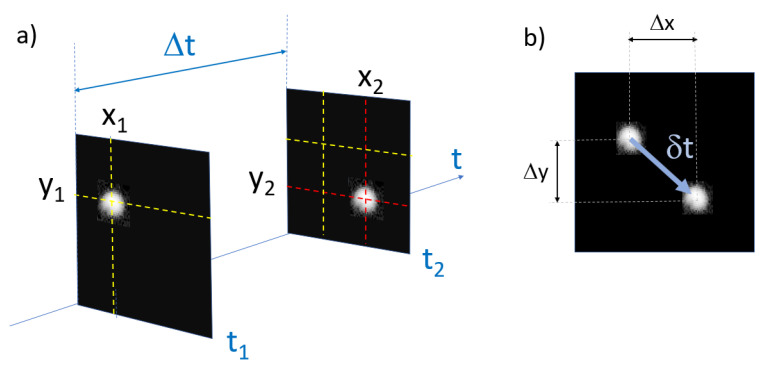
Description of the variation of the scanning time between two successive positions. (See Section S3 of Supplementary Materials for complete description of the timing projection for image construction.) (**a**) $X_{1}$ and $Y_{1}$ represent the position of the particle in the frame 1 and $X_{2}$, $Y_{2}$ its position in the frame 2. $t_{1}$ represents the time needed to scan every pixel of each line to reach the particle in the first frame and $t_{2}$ is the same for the second frame. (**b**) The particle with a movement in the direction of the scan will add or delete a time depending on the sign of $\delta t=t_{2}-t_{1}$ to the normal time Δt to complete a full scan of one frame.

Compared to the common SPT method, using a scanning method for tracking causes the lag time to be only approximately constant between frames. Indeed, the movement of a particle between two images adds or removes some travel time for the laser to scan the next position. This difference of travel in the scan causes a variable time interval between each frame. Every displacement has a unique time lag depending on the direction of the movement between two successive positions as described in Figure 4.

In this study, two approaches will be used: the first is the common method assuming a constant time between two successive frames called time lag which represents the time needed to create an image and come back to the first pixel. The second method uses a specific time based on the periodic time lag of the scan but also takes into account the displacement of the particles between two frames.

In the approximation of a constant time lag, the characteristic time is called Δt:

(1) $$\Delta t=N_{pix}^{2}\;t_{dwell}+N_{pix}\;t_{dwell}\;\;\;\left[s\right]$$

$N_{pix}$ is the number of pixels per line and $t_{dwell}$ is the dwell time (the NLO signal is collected during the dwell time of the beam on the sample for each position). The second term of Equation (1) represents the time needed for the laser to travel back to the first pixel of the image as shown in Figure 3 with the red arrow.

For the variable time lag, the characteristic time τ is defined as follows:

(2) τ=Δt+δt[s]

with δt the time to add or subtract due to the displacements detailed in Figure 4:

(3) $$\delta t=\Delta Y_{pix}\;N_{pix}\;t_{dwell}+\Delta X_{pix}\;t_{dwell}\;\;\;\left[s\right]$$

with $\Delta Y_{pix}$ and $\Delta X_{pix}$ the number of pixels displacement on each direction. Because of the scanning method, a displacement along Y or X is not equivalent in a matter of time travel. A displacement on the Y axis requires a full scan of every previous line, whereas for an X axis displacement only needs to scan the previous pixel from the beginning of the line. To simplify Equation (3), it is possible to approximate δt by the first term:

(4) $$\delta t=\Delta Y_{pix}\;N_{pix}\;t_{dwell}\;\;\;\left[s\right]$$

Indeed, according to the chosen scanning method, the time depending on the X movement is not relevant, first because the scan changes direction at every line change and secondly, for the same number of pixels displacement, $\delta t_{y}>>\delta t_{x}$.

### 2.6. Tracking

**Figure 5:**
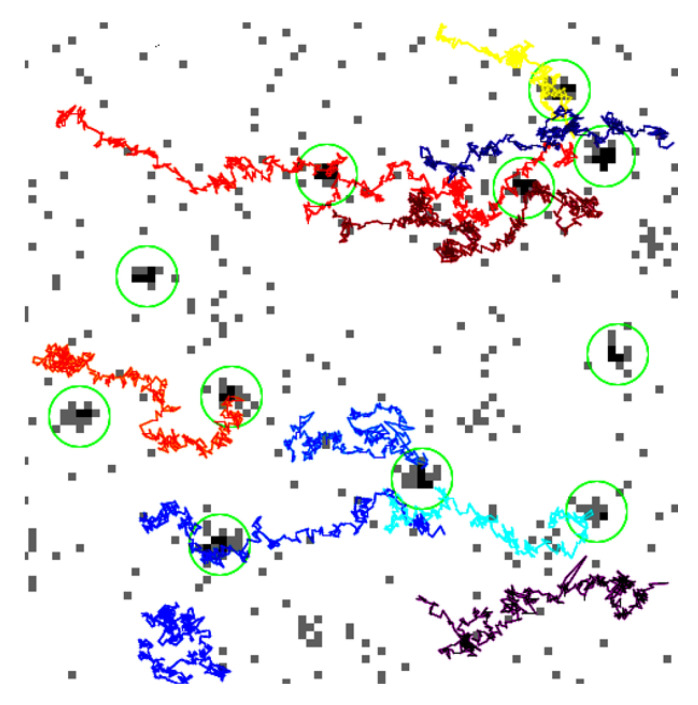
A few tracks recorded over 600 frames over an area of 52 × 52 μm with the plug-in “TrackMate” of ImageJ developed by J.Y. Tinevez. The detected particles are circled in green and their tracks are marked using different colors. The isolated grey spots are some noise from the PM and are not detected as particles according to a minimum detection size.

The powerful tool called TrackMate developed by J.Y. Tinevez is used to process the tracks of particles (see Figure 5) in image sequences [27]. The chosen method for the localization is the LoG (Laplacian of a Gaussian) detector which applies a LoG filter to the image and achieves a maxima localization with a sub-pixel precision thanks to a quadratic fitting. Then successive frames are examined to find a link between particles according to a maximum search radius which corresponds to the maximum distance a particle could have moved between two frames. The algorithm can also account for particle disappearance over a small number of frames (detection error or moving out of focus). Finally, the full track is built by assembling all the successive positions for each particle. The Sections S4–S6 of Supplementary Materials describes the raw acquired images, the TrackMate process and used algorithms.

## 3. Estimator

### 3.1. Theory

**Diffusion coefficient estimation:** To link displacement of the particle to its size, Einstein established the relation between the Brownian motion and the diffusion coefficient with the mean square displacement (MSD) [2]:

(5) $$<\Delta x^{2}>=2dDt\;\;\;\;\left[m^{2}\right]$$

where *D* is the diffusion coefficient [m${}^{2}$/s], *d* the number of dimensions of the movement and *t* the duration of the observation [s].

The Stokes-Einstein law links the diffusion coefficient *D* and the parameters of the experiment. It is used to obtain the size of spherical particles:

(6) $$D=\frac{k_{b}T}{6\pi \eta r}\;\;\;\;\left[m^{2}/s\right]$$

with $k_{b}$ the Boltzmann constant, *T* the temperature in Kelvin, η the dynamic viscosity of the fluid in Pa.s and *r* the hydrodynamic radius of the particle in meters.

### 3.2. Experimental Estimation

The previous Equation (5) gives the theoretical diffusion behavior of spherical particles, but may not be the best approach to extract the diffusion coefficient from experimental data. Several methods have been developed to optimize the estimation according to experimental parameters. Here, different methods are presented, two of them are known and the last one is adapted to non-linear microscopy.

**Mean Square Displacement:** The most basic and common method is to calculate the mean square displacement [28] for different time lags and establish the slope coefficient of the fitted curve to get the diffusion coefficient. In practice, only the first two points are used to evaluate the diffusion coefficient. Some studies show that the error is correlated with the frame rate used to estimate the slope [29]. It is also shown that using short or long time lag does not improve the accuracy of measurements overall. Moreover, in the present case, the small amount of data makes the use of coefficients based on longer time lags unreliable at best.

(7) $$D_{n}=\frac{<\Delta x^{2}>}{2dn\Delta t}\;\;\;\;\left[m^{2}/s\right]$$

where *n* represents the number of time lags between two frames (n = 1, 2, 3,...). For example, in the calculation of *D1*, Δx is evaluated between all the successive positions, while for D2, Δx is evaluated between positions separated by one frame, and so on.

**Covariance-based estimator:** A new estimator elaborated in 2014 [9] is unbiased and more accurate than the previous method when the S/N is low and the number of positions is close to 100. The covariance estimator is expressed as follows:

(8) $$D_{CVE}=\frac{<\Delta x_{i}^{2}>}{2d\Delta t}+\frac{<\Delta x_{i}\Delta x_{i+1}>}{d\Delta t}$$

with $\Delta x_{i}=x_{i}-x_{i-1}$, where *x* is the position and *i* the frame number. The second term of the sum represents the localization noise.

The same equation can be written according to experimental parameters, depending on the microscope characteristics, as follows:

(9) $$D_{CVE(\sigma ,R)}=\frac{<\Delta x_{i}^{2}>-2d\sigma^{2}}{2d(1-2R)n\Delta t}$$

where σ is the dynamic localization error (see Section 5.1) and *R* the motion blur coefficient [30]. *R* is not estimated in the usual way [see Equation (13)] because of the scanning process as explained further (see Section 4.2).

**Variable time lag estimator:** A new method is tested in this experiment because of the scanning process and the variable time lag. Instead of using the mean square displacement, a diffusion coefficient is calculated for every displacement with its own time lag, then a mean diffusion coefficient is evaluated.

Let $<D_{n}>$ be the diffusion coefficient calculated with different time lags:

(10) $$<D_{n}>=\langle \frac{\Delta x_{i}^{2}}{2dn\tau }\rangle$$

with τ the variable time between two successive positions as described in Equation (2).

For the covariance-based estimator with the variable time lag τ, the Equation (9) changes as follows:

(11) $$<D_{CVE(\sigma ,R)}>=\langle \frac{\Delta x_{i}^{2}-2d\sigma^{2}}{2d(1-2R)n\tau }\rangle$$

## 4. Overview of Improvements and Limits with NLO Microscopy

To properly understand the problem, we need to redesign several aspects of single-particle tracking with single-photon microscopy. As we saw previously, the time lag changes radically; however, several other parameters must be modified as well.

Using non-linear microscopy is a very different way to create an image of a sample because of the scanning step. It already takes a long time to create a low resolution image (30–50 ms for 100 × 100 pixels) and contrary to conventional NLO imaging it is not possible to accumulate images in order to increase the number of detected photons because of the particle motion. The following parts deal with the advantages of using non-linear imaging on moving particles which cannot be obtained by other microscopy techniques.

### 4.1. Photobleaching

The photobleaching effect is an alteration of the photochemical property of the fluorophore molecule [31]. This phenomenon limits the duration of the tracking when all the fluorescent molecules are fully damaged. In both single- and two-photon microscopy, this effect appears because of the high laser intensity. However, when dealing with a large amount of particles in a free diffusion movement, the small focal volume and scanning process of two-photon microscopy are more advantageous. When a wide-field image is made with single-photon excitation microscopy, a large and deep area is illuminated, and many particles are subjected to the photochemical process. On the contrary, two-photon microscopy, which is intrinsically confocal due to the non-linear absorption process, excites only a smaller volume for a very short time with stronger illumination to fully record the signal of the particle. Compared to one-photon absorption, a two-photon process reduce photobleaching because out-of-focus particles are illuminated by the laser but not excited.

### 4.2. Motion Blur

**Figure 6:**
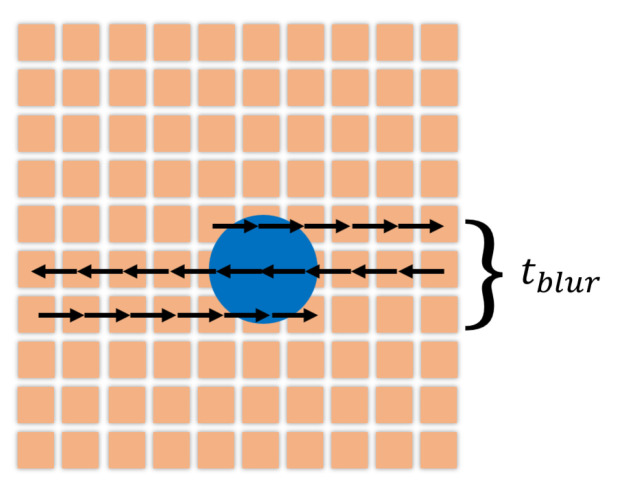
Description of motion blur with scanning process. $t_{blur}$ is the time spent to achieve the scan of the object. The blue circular shape (blob) represents the particle subjected to scanning and the arrows are the path to fully scan the particle.

Motion blur is a phenomenon appearing when the object recorded is too fast for the exposure time of the camera and causes the signal to spread over an area around the object. However, motion blur is less significant with the scanning process described in Figure 3. Even if the full image takes longer to be acquired, the time spent scanning the object is really short (5–10% of Δt) as described in Figure 6:

(12) $$t_{blur}=S_{blob}\;t_{dwell}\;N_{pix}$$

with $S_{blob}$ the size in pixel of the round point spread function (PSF) due to the particle on the image. To calculate the motion blur coefficient, this formula is usually used [30]:

(13) $$R=\frac{1}{\Delta t}\int_{0}^{\Delta t}S\left(t\right)\left(1-S\left(t\right)\right)dt$$

with S(t) the aperture function of the camera representing the fraction of illumination happening before a time *t*. More precisely $S\left(t\right)=\int_{0}^{t}\varsigma \left(t^{\prime }\right)dt^{\prime }$ with ς(t) the normalized state of the camera shutter during a tracking frame (ς(t) = 0 means closed shutter, while (ς(t)>0 means open shutter.). The common values of *R* are in the interval of 0 to 1/4 (with R=1/6 for a shutter open during the full duration of a tracking frame). For a non-linear scanning process, we can consider that the camera is only open during the time $t_{blur}$ (cf Figure 6). The motion blur coefficient is then easily calculated:

(14) $$R=\frac{t_{blur}}{\Delta t}$$

and common experimental value is here R≈0.06.

### 4.3. Brilliance and Numerical Aperture

To optimize an estimation with SPT, it is necessary to record as many positions as possible for each particle. Knowing that non-linear microscopy has a very small axial excitation volume compared to conventional one-photon microscopy, there is a small probability for every particle to stay in this excitation volume for a long period of time. Consequently, the best objective is not the one with the highest numerical aperture (NA), which would lead to an even smaller axial focal volume. A low NA is not satisfying either, because the laser spot must be focused enough to generate NLO effects. A compromise had to be found between these two factors, and was achieved through a X20, 0.75 NA objective. The numerical aperture of the objective can be used to estimate how long a particle can stay in the excitation volume. The two-photon excitation depth can be calculated as follow [32]:

(15) $$w_{z}=\frac{0.532\lambda }{\sqrt{2}}\left[\frac{1}{n-\sqrt{n^{2}-NA^{2}}}\right]$$

with λ the wavelength of the excitation laser, n the refractive index and NA the numerical aperture of the objective. To estimate an average track duration $t_{vox}$, the mean square displacement (Equation (5)) can be assimilate as the voxel depth $w_{z}$ [1]:

(16) $$t_{vox}\approx \frac{w_{z}^{2}}{2D}$$

However, one can note that in this experiment, the NA of the objective is not fully used. The laser beam diameter is only 4 mm over a back-aperture diameter of 14 mm. One can estimate the effective numerical aperture via 4/14×0.75≈0.2 which was also confirmed with numerical simulations (see Section S7 of Supplementary Materials) An effective NA at around 0.2 for the excitation leads to an axial voxel excitation depth $w_{z}$ of around 15 μm. This configuration has been maintained because the microscope uses several objectives with different back-aperture sizes. This is an important aspect to consider because the excitation light is not as focused by the objective as it could be. This increases the voxel depth while reducing light intensity for the two-photon excitation. On the other hand, because the light collected from the sample enters through the front face of the objective, the full NA must be taken into account for the collected signal leading to more photons being collected. As a side note, the axial detection volume size is not limited here by the depth of field of the objective. Indeed, the size of the active area of the photomultiplier (around 5 mm) leads to a large geometrical optical circle of confusion and a depth of field around 300 μm. Consequently, the axial detection volume size is here determined by the excitation profile and is around 15 μm. Finally, we use a dry objective for measurements within water which could increase the spherical aberrations and, as a result, increase the size of the observation volume. In order to limit those effects, we work at around 20 μm above the surface so that on one hand, the surface does not alter the free Brownian motion and, on the other hand, the mismatch of indexes does not create too many aberrations.

### 4.4. Image Resolution and Frame Rate

Considering that the estimation requires a large amount of data, the frame rate of the sequence acquisition is really important to ensure that enough data have been recorded before the particle leaves the focal volume. However, this frame rate setting influences all the other image quality parameters. So to optimize the amount of data, it is again important to find a compromise between resolution and frame rate.

### 4.5. Image Resolution and Localization Error σ

Another important aspect of the estimation is the static localization error which sets the accuracy of the measurement. In other words, generating an image sequence of static particles will lead to a false detection of movements because of the fluctuation of the pixel value. Several other parameters, such as the intensity of the signal emitted by the fluorophore and the detector noise, are linked with the localization error. However, it was previously established that the image resolution and the localization error are linked with the frame rate and again, a compromise had to be found.

### 4.6. Scanned Area and Image Resolution

The last relevant parameter is the artificial zoom of the scanning step. For the same number of pixels, if a smaller area is scanned, it leads to a zoom. Then, more pixels are used to depict the particle, but the scanning duration remains the same, causing the localization error to decrease. Estimating a center over many pixels is always easier than over a few. The zoom is the best tool to increase the image resolution without reducing the frame rate. Nevertheless, this tool has a limitation: a large part of the image needs to be empty so that the movement of the particle can be tracked.

### 4.7. Signal-To-Noise Ratio Localization and Particle Size

The $SNR_{loc}$ [9] is often used to assess the quality of a measurement and the highest possible accuracy:

(17) $$SNR_{loc}=\frac{\sqrt{D\Delta t}}{\sigma }$$

with, *D* the diffusion coefficient [m${}^{2}$/s], Δt the time lag between two frames [s] (for a variable time lag, <τ>=Δt) and σ the static localization error [m].

Typically, 1 <$SNR_{loc}$< 20 in SPT experiment, but in our case, $SNR_{loc}$≈ 2.5 because of the high localization error (cf Section 5.1: Static and dynamic localization error). However, the $SNR_{loc}$ is almost constant thanks to a balance between the size of the particle and the zoom. Indeed, the motion of a particle depends on its size: a small one moves faster than a big one, so its displacement between two frames increases. On the other hand, reducing the zoom is necessary to record all its movement, thus the localization error increases as well.

## 5. Results and Discussion

This part introduces the results obtained for single-particle tracking of fluorescent beads of two different sizes with the scanning microscope described above. Numerous tests showed that the best compromise between frame rate and image resolution for the setup is a 100 × 100 pixels image. Its characteristics are detailed in Table 1. Increasing the frame rate is not possible due to the limited scanning speed of the two-axis scanning head (max speed 3μs/pixel), and the number of positions recorded before the particle exits the excitation volume would be too low if the frame rate is decreased.

### 5.1. Errors

To estimate the size of the particles, many steps are involved and each of them adds a possible error. We will try to estimate most of these before showing the size results.

Size distribution: Every sample of fluorescent beads is manufactured around a specific radius, with a dispersion characterized by the standard deviation, 5% for the 250 nm radius beads and 3% for the 110 nm radius beads.

Sampling error: Every statistical method of estimation using only a sample of the population leads to a fractional sampling error [33], defined by:

(18) $$\epsilon =\sqrt{\frac{2}{N-1}}$$

This equation shows that a small amount of data positions, around 100, produces a 14% sampling error on the estimated value.

**Figure 7:**
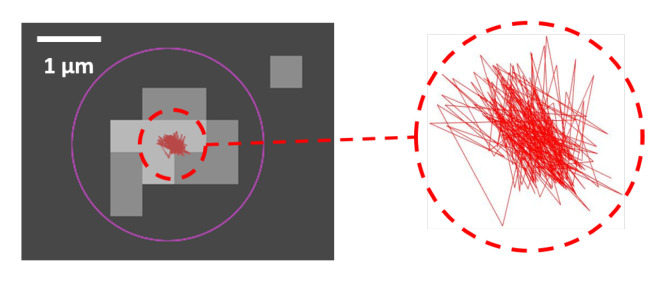
Track detected by the algorithm for a particle of d<1μm at rest on a coverslip glass for an image of 100 × 100 pixels, 1.9 pixel/μm over 200 frames. The track is represented by the small red pattern in the center and enlarged in the dotted circle (right).

Static and dynamic localization error [34]: To assess the accuracy of the localization, two parameters are involved. First, the resolution and the dynamic range of the image with its number of pixel and the number of collected photons. Secondly, the quality of the algorithm used to evaluate the positions in the image. A suitable method to assess the overall localization accuracy entails using a particle with a size under the diffraction limit deposited on a substrate at rest, and to measuring the track detected by the algorithm. Typical variations in the position of detected static particles are shown in Figure 7. These fluctuations are attributed to residual vibrations (e.g., from the microscope table), non-linear signal and detector fluctuations, inaccuracies in the position of the scanning mirrors or errors in estimating the center of the particle by the TrackMate algorithm. For the selected parameters, the static localization error amounts to $\sigma_{0}\approx 80$ nm (std=40 nm) for 250 nm radius beads and $\sigma_{0}\approx 100$ nm (std=50 nm) for 110 nm radius beads.

When the particle is moving, this error becomes a dynamic localization error, based on the previously measured $\sigma_{0}$ as follows [25]:

(19) $$\sigma =\sigma_{0}\sqrt{1+\frac{D\;t_{dwell}}{s_{0}^{2}}}$$

with $\sigma_{0}$ the static localization error, D the diffusion coefficient, $t_{dwell}$ the scanning time on the particle and $s_{0}$ the radius of the PSF. However, for the selected scanning method, the exposure time is very short, therefore the dynamic error can be approximated by (±1 nm) the static error $\sigma \approx \sigma_{0}$.

### 5.2. Size Estimation

The goal of this experiment is to evaluate the dispersion of the size estimation for the two samples of fluorescent beads. To increase the probability of tracking a single-particle over more than 100 positions, it is necessary to scan many frames. From Equation (16), for a 250 nm radius bead and a 1 μm voxel depth, the particle theoretically exits the volume after 0.6 s. Considering this, the average track has 12 positions at 20 fps, but the underfilled back-aperture of the objective allows a deeper focal volume. The track length can be increased using a concentrated solution of beads to leverage the Gaussian distribution of track lengths. Only the tracks comprised of over 100 positions are then selected.

To evaluate the experimental performance of the previous methods, a radius (Rx,y) is estimated for both axis, *x* and *y* with the diffusion coefficient (see Section 3.2) and $R_{z}$ is the average of $R_{x}$ and $R_{y}$. When it is possible, the diffusion coefficient is calculated with the two different approaches, the constant and variable time lags. The different estimators are compared according to their efficiency for non-linear microscopy.

The mean square displacement slope (MSD) can only be evaluated with a constant time lag Δt. The mean square displacement cannot be calculated using the variable time lag τ because each displacement is evaluated with a different time lag.

The estimation of the diffusion coefficient can be calculated with the standard estimator (Equation (7)) for two different time lags, $D_{1}$ for Δt and $D_{2}$ for 2Δt. It shows the behavior of the method when the constant time lag increases. Those two coefficients can be estimated for variable time lags ($<D_{1}>$ and $<D_{2}>$) to evaluate the influence of this method. When the coefficients are estimated with different time lags it means the first coefficient $D_{1}$ is calculated with the native time lag of the image sequence Δt, whereas $D_{2}$ uses separated odd and even images to have 2Δt.

The last chosen way to estimate the diffusion coefficient is the covariance-based estimator with its two different formulations, $D_{CVE}$ (Equation (8)) with the localization noise term and $D_{CVE(\sigma ,R)}$ (Equation (9)) with the experimental parameters from the microscope. To evaluate this estimator for a variable time lag, only Equation (9) can be used. Indeed, Equation (8) cannot be adapted to this method because it takes into account the displacements $x_{n}$ and $x_{n+1}$ which have different time lags; thus this equation would require a new formulation.

The results obtained with the different methods are shown in Table 2, with the mean radius and the standard deviation estimated in the sample. Between the 250 and 110 nm radius beads, it is necessary to reduce the dwell time (cf Table 1 in order to record enough data for each track (data > 100 positions) because of the higher velocity of smaller particles. However, reducing the dwell time decreases the number of photons detected by the PM and thus the S/N, which is why the zoom is increased to enhance the S/N. The excitation wavelength of the laser is 760 nm for beads of both sizes.

**Method performance:** To evaluate the experimental performance of the methods, two indicators are important: the mean value and the standard deviation. The best performance for the mean value is achieved using $D_{CVE(\sigma ,R)}$, as expected according to the method’s performance discussed previously [9]. The estimation of the diffusion coefficient using only the first time lag ($D_{1}$) shows good results with a lower dispersion than for the covariance-based estimator, but causes the mean to shift slightly. Both $D_{CVE}$ and MSDs result in unreliable estimations of the mean and significant dispersion across all the measurements. It seems that on specific tracks with singular behavior, those two methods are really sensitive and completely overestimate the size with a radius measured around 500 μm. However, $D_{CVE}$ seems to improve when the frame rate increases for the 220 nm beads.

**Figure 8:**
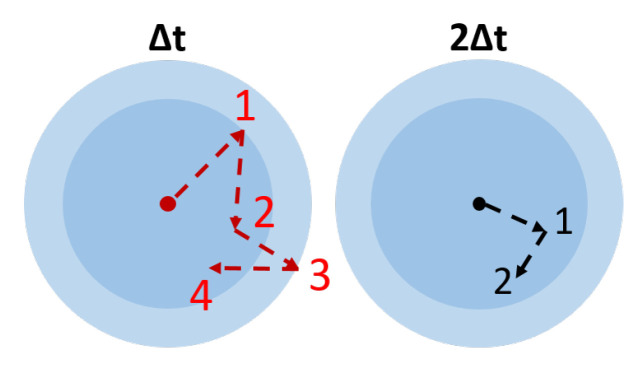
Representation of the behavior of a Brownian movement seen with two different acquisition times [34]. The center dot represents the particle and the arrows are the displacements detected. The same track is represented with two different observation time lags, on the left Δt and on the right 2Δt. The shaded circles represent the diffusion area where a particle should be found after a diffusion time of Δt (first circle) and 2Δt (second circle) if we take as radius the square of Equation (5).

An interesting evolution can be observed with $D_{1}$ and $D_{2}$ coefficients depending on the time lag used for the estimation of the diffusion coefficient (see Figure 8). As the time between two frames increases, the accuracy on the coefficient worsens, the size being increasingly overestimated. Given that the $SNR_{loc}$ is higher when time lag is longer, it could be assumed that the estimation would improve but the exact opposite is true. Since the total lapse of time during which the particle can be observed is limited (the particle ends up exiting the volume), increasing the time lag will decrease the total amount of data available for the calculation of the coefficient D, thus significantly decreasing the accuracy of the estimation.

The last aspect of the results is the effect of the variable time lag called τ previously. It seems that the modification brought by this new way of estimation does not affect the final result significantly. Even if its use seems to be justified, no real influence is observed, and it can be explained by the localization error of the setup. Referring to the experimental data for 250 nm radius beads, the maximum displacement $h_{max}$ observed is 1.14 μm, while the theory [35] gives:

(20) $$h_{max}=3\sqrt{\pi D\;t}=1.11\;\mu m$$

Then, we can consider the extreme case where this displacement only happens in the linear scanning direction and adds an extra scanning time:

(21) $$\delta t_{max}=h_{max}\;N_{pix}\;t_{dwell}\;R_{ima}=110\;\mu s$$

With $R_{ima}$ the scale resolution of the image. Here $R_{ima}$ = 2.53 pixel/μm, $N_{pix}=$ 100 pixels and $t_{dwell}=5\;\mu$s. Knowing this extra time produced by the displacement, we can evaluate the difference on the diffusion length over $\delta t_{max}$:

(22) $$h_{gap}=h_{max(t=\Delta t+\delta t)}-h_{max(t=\Delta t)}\approx 12\;\mathrm{nm}$$

Comparing this result which covers all the extremes cases (with a low occurrence) with the static localization error of the measurements, $h_{gap}<\sigma$, we can say that using a variable time lag for the estimation is insignificant if the localization error is five time higher than the extra accuracy brought by the new method.

## 6. Conclusions

In conclusion, this study shows the feasibility of tracking particles in liquid phase using non-linear microcopy and punctual detector. It can provide a robust estimation of a diffusion coefficient. As described, this study is mainly experimental and required several attempts, various materials, and different configurations of the setup to find a good compromise between localization accuracy and frame rate. The coefficient $D_{1}$ provided good results but is sensitive to the chosen frame rate. Finally, only $D_{CVE\left(\sigma ,R\right)}$ can be considered a good estimator according to the results and can be easily adapted to non-linear microscopy with the specific parameters σ and *R*. The improvement brought by considering the variable time lag will be useful in the future if the localization accuracy is increased, but has no impact at the moment. Improvements in this technique are possible. A way to improve the accuracy of the measurements is to reduce the localization error thanks to a better objective with higher numerical aperture. This solution is only possible if the particles size makes it possible to record enough data, otherwise a compromise is needed between localization accuracy and track length. Another way to improve the accuracy of the measurements is to refine a new particle localization algorithm suitable for images with low resolution and photon signal. 

## Figures and Tables

**Table 1:** Optimized parameters for tracking fluorescent beads with TPEF scanning microscopy

Particle	Image Size	Dwell Time	Frame Rate	Scale
Radius (nm)	(pixels)	(μs)	(fps)	(pix/μm)
250	100 × 100	5	20	2.53
110	100 × 100	3	33	3.81

**Table 2:** (**A**) Table of the radius $\left(R_{x,y}\right)$ estimated for the 250 nm of radius beads with all different methods. Results calculated over 43 trajectories with a minimum of 100 positions each and an uncertainty on the radius evaluated at ±55 nm. (**B**) Table of the radius $\left(R_{x,y}\right)$ estimated for the 110 nm of radius beads with all different methods. Results calculated over 25 trajectories with a minimum of 100 positions each and an uncertainty on the radius evaluated at ±32 nm.

(A) *r* = 250 nm	MSD	$D_{1}$	$D_{2}$	<$D_{1}$>	<$D_{2}$>	$D_{\mathrm{CVE}(\sigma ,R)}$	$<D_{\mathrm{CVE}(\sigma ,R)}>$	$D_{\mathrm{CVE}}$
Mean radius $R_{x}$ (nm)	411	252	308	253	308	269	270	418
$\sigma (R_{x})$ (nm)	115	46	58	46	58	58	58	127
Mean radius $R_{y}$ (nm)	725	221	325	221	325	230	231	746
$\sigma (R_{y})$ (nm)	439	41	69	42	68	51	51	507
$R_{z}$ = ($R_{x}$+$R_{y}$)/2 (nm)	568	237	316	237	316	250	250	582
**(B) *r* = 110 nm**	**MSD**	$D_{1}$	$D_{2}$	**<** $D_{1}$ **>**	**<** $D_{2}$ **>**	$D_{\mathrm{CVE}(\sigma ,R)}$	$<D_{\mathrm{CVE}(\sigma ,R)}>$	$D_{\mathrm{CVE}}$
Mean radius $R_{x}$ (nm)	143	101	116	101	116	105	106	160
$\sigma (R_{x})$ (nm)	31	15	13	15	13	19	19	43
Mean radius $R_{y}$ (nm)	286	90	131	90	131	92	92	90
$\sigma (R_{y})$ (nm)	220	12	25	12	25	14	15	12
$R_{z}$ = ($R_{x}$+$R_{y}$)/2 (nm)	214	96	124	96	123	99	99	125

## Supplementary Materials

The following are available online at https://www.mdpi.com/2079-4991/10/8/1519/s1, Section S1: Optical Setup, Figure S1: Optical optimization of laser beam. Figure S2: Complete setup of the non-linear microscope. Section S2: Raster scan methods, Figure S3: Illustration of used Raster scan methods. Section S3: Timing of images, Figure S4: Timeline of acquisition of successive images and lag time between images. Section S4: Raw images, Figure S5: Raw image of latex beads of 500 μm of diameter in a droplet of water. Figure S6: Histogram of the raw image. Section S5: TrackMate process, Figure S7: Procedure to generate coordinates of particles from TrackMate. Section S6: Algorithm, Figure S8: Flow chart of the algorithm for constant time lag. Figure S9: Flow chart of the algorithm for variable time lag. Section S7: Calculation of focal volume size via vectorial diffraction.
