# Supplementary material for: Single-Particle Tracking with Scanning Non-Linear Microscopy
Source: Nanomaterials (Basel). 2020 Aug 3;10(8):1519. doi: 10.3390/nano10081519 (PMC7466504; doi:10.3390/nano10081519)
Supplement: Supplementary file 1 [file nanomaterials-10-01519-s001.pdf]

Supplementary Materials: Single-Particle Tracking with Scanning Non-Linear Microscopy

Théo Travers , Vincent G. Colin , Matthieu Loumaigne, Régis Barillé and Denis Gindre*

1. Optical setup

This study was carried out with an inverted scanning microscope. The optical optimization of laser beam is illustrated in Figure S1. The general diagram of the setup is shown in Figure S2.

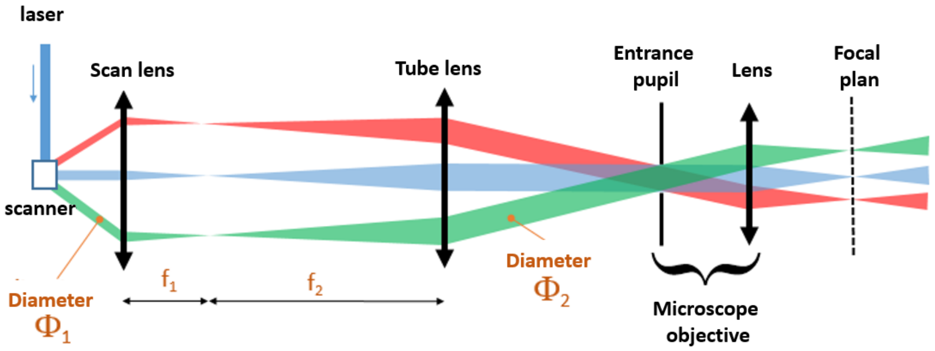

Figure S1. The laser beam is deflected by oscillating mirrors (scanner). Two lenses are used to image the scanning device to the back aperture of the microscope objective. The lens after the scanner is the scan lens with a focal length f1, while the other lens is the tube lens with a focal length f2.

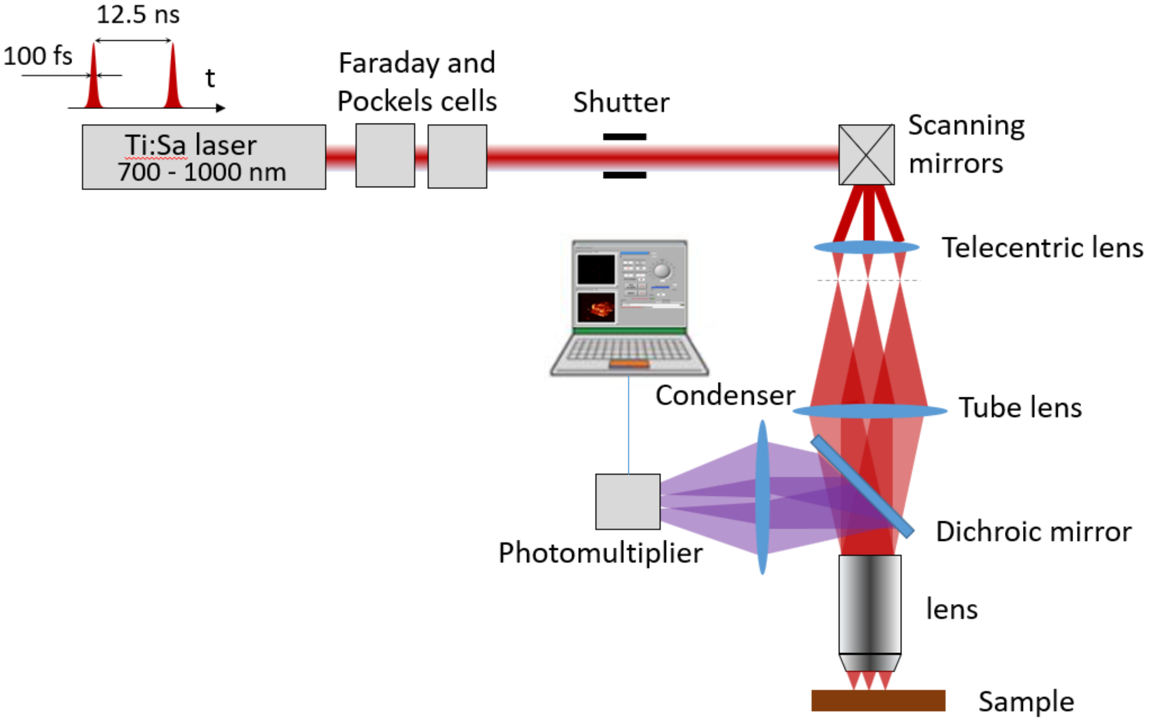

Figure S2. Complete setup of the nonlinear microscope, which can be used for two photons fluorescence (TPF) and second harmonic generation (SHG).

The laser is a Ti:Sa femtosecond laser (Tsunami, Spectra-Physics) pumped by a 10 W continuous YAG:Nd laser at 532 nm (Millenia, Spectra-Physics). The maximum gain is obtained for = 750 nm

(average emission power 1.4 W). The pulse duration is about 80 fs at the laser output, but after passing through various optical elements, the chromatic dispersion widens each pulse whose effective width is estimated at 100 fs at the sample level. The rate of the laser is 80 MHz.

The laser beam first passes an electromechanical shutter with an automated control, (Uniblitz). An optical fiber is used to take part of the beam scattered on the shutter and to direct it to a compact spectrometer (OceanOptics) in order to visualize the emission spectrum on a dedicated computer, via the OOIBase32 software. The spectral width and the emission wavelength are thus controlled.

The laser beam passes successively through two cells: a Faraday isolator (IO-3-780-HP, Thorlabs) and an ultra-fast Pockels cell (350-80LA, Conoptics). The first cell is designed to prevent any reflection from returning to the laser cavity. The electrically controlled Pockels electro-optical cell is used as an intensity modulator. A half-wave plate $\lambda/2$ achromatic (700–1000 nm, Edmund Optics) placed on a motorized rotation stage (PR50, Newport) controls the beam polarization. This stage is connected to one of the eight power controls of a universal controller (XPS, Newport).

The study area is scanned using two galvanometric mirrors (6210H, Cambridge Technology Inc.) controlled by a two-axis driver (MicroMax, Cambridge Technology Inc.). These drivers are voltage-controlled via a signal delivered by an analog card at a maximum rate of 1 MHz and coded on a 12-bit dynamic range (NI PCI-6711, National Instrument). The swept angular space at the output of the mirrors, and by extension the swept area on the sample in the focal plane of the microscope objective, is directly related to the amplitude of this voltage. The different apertures encountered in the microscope strongly limit this amplitude, which must be limited to 2V peak-to-peak in order to obtain a uniform distribution of the laser intensity over the entire scanned surface.

The microscope used is a commercial inverted microscope (IX71, Olympus). A collimated green LED source (Model MTN005303, Thorlabs) is used to make images of the sample in brightfield reflection with a high-sensitivity monochrome CMOS camera (UI-3240ML, Stemmer Imaging). The specimen holder is placed on a motorized positioning stage (XYZ 562-ULTRALIGN and 3 TRA12CC motorized stages, Newport Corp.). The laser beam that is directed onto the microscope is a beam previously deflected by the scanner. An afocal optical mount provides the desired beam broadening factor. The chosen solution is to use a scanning lens placed according to the diagram in Figure S2. In order to reduce geometrical aberrations, a telecentric lens with a focal length of 54 mm (LSM54-850, Thorlabs) was finally implanted.

The reflected beam (SHG or TPEF) is directed onto an ultra-fast photomultiplier (H7422P-40, Hamamatsu) with a rise time of 1 ns and a spectral response ranging from 300 nm to 720 nm, the maximum sensitivity being at 580 nm for a quantum efficiency of 40%. The digitized signal (TTL pulses) is then directed to a photon counting module (C9744, Hamamatsu) with a counting frequency of up to 10 MHz. This module is connected to one of the 8 32-bit counters of a high-speed PCI card (PCI-6602, National Instrument). The timing of the counting module is synchronized with the electronic signal controlling the scan mirrors by a specific high-speed RTSI (Real-Time System Integration, National Instrument) bus through a cable that directly connects the two PCI cards to each other.

The control interface was developed in our laboratory with the Labview design platform (LabVIEW 2010, National Instrument). This interface controls the positioning boards, the laser intensity and polarization, the scanning parameters, it generates the scan signals and the images from the non-linear optical signal obtained by the counting module, performs the synchronization between these two events (scanning and detection) and can save the obtained images as well as all the parameters used for the acquisition.

The scanning settings are as follows:

- Image size. The program was developed to generate square images. Therefore, choosing 100 dots for a line generates an image of 100 x 100 = 10,000 pixels.

- Zoom factor: This is the maximum voltage applied to the scan mirrors, which determines the physical size of the scanned area of the sample.

- Exposure time: This is the dwell time, that is the integration time during which the counting takes place. This time corresponds to the laser exposure time of each scanned area of the sample. The minimum value of the exposure time, imposed by the mechanical movement of the mirrors, is 1 μs.

2. Raster scan methods

Several methods have been tested to scan the area of interest. The scanning imaging particle tracking method requires the optimization of the scanning time to obtain high frame rates. For this purpose, the sampling rate of the electronic counter is set to a value close to 1 MHz, which corresponds to a time-laps close to 1 microsecond, including the dwell time and the time for moving the mirrors to the next position.

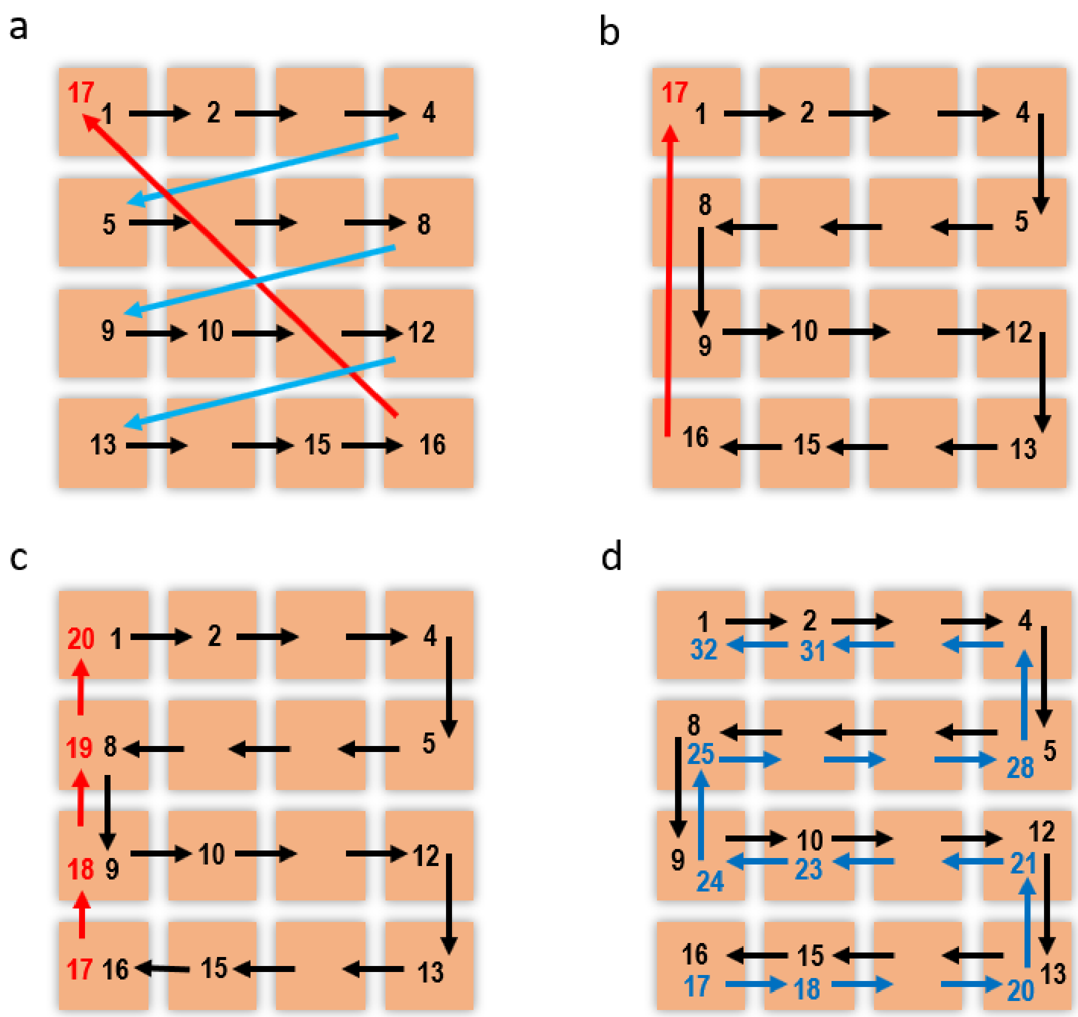

Figure S3. (**a**) Traditional Raster Scan. (**b**) Traditional Snake Scan (continued raster scan). (**c**) Snake Scan with controlled return (dead) time. (**d**) Snake scan back and forth without dead time.

With a traditional raster scan (Figure S3a), all lines are scanned in the same direction. This requires the "column" mirror to return very quickly to the beginning of the line when the last position on the

previous line has been reached. For fairly large scanned areas, this return time may be longer than the sampling period. In this case, the image obtained is not representative of the scanned area because measurements were taken during the return.

With a "snake" scan (Figure S3b), travelling back to the beginning of the line is no longer an issue because the lines are scanned alternately in one direction and in the other. This method requires to reconstruct the final image by inverting the even lines, but it has the huge advantage of eliminating dead times after each scanned line. However, there is still a dead time at the end of the scan, which allows the mirrors to return to the initial position for the next scan. Although this dead time is usually not an issue, it is directly linked to the frame rate in the method described here. The issue with this direct return to the initial position is that the time needed to complete the movement is not exactly known. Indeed, for small scan areas, this time will render only a few sampling periods, while for larger scan areas, the number of sampling periods taken up by this displacement increases.

The final solution chosen is represented in Figure S3. It is a snake-like raster scan, but the return to the initial pixel is controlled, by scanning pixel by pixel the first column from the bottom to the top. This dead time is therefore controlled, and its value is perfectly known for each acquisition. Still, it remains a dead time, corresponding to the scanning of an additional column. Because this time is perfectly known and controlled, the exact frame rate is then perfectly known for the future size estimations.

The Figure S3d represents a scanning method which was tested and eventually abandoned. Its purpose is to reduce as much as possible the dead time between each image, by making two successive inverted images with a snake-like scan. With this approach, once the area has been scanned the first time, the mirrors take the reverse path to return to the starting point. A sequence provides the data to create two images, with maximal frame rate and no dead time. This method is the most efficient to obtain quickly images of still objects, but it does not work for the present goal because the time to access a given particle is not the same for even and odd images, making further processing for tracking unnecessarily complicated.

3. Timing of images

In nonlinear microscopy the illuminating beam should scan an area in order to build up the image pixel by pixel. As a result, each image is not a snapshot of the observed scene, because each pixel is acquired at different times. This is the fundamental difference from the usual particle tracking techniques based on video sequence analysis.

The photons emitted by the fluorescent particles are captured by a photomultiplier tube. Consequently, the information about each pixel is acquired at different times corresponding to the dwelling time per pixel (the time spent by the laser to scan each pixel).

Snake-shaped raster scanning means that no time is wasted by alternating the scanning direction of the lines. A square image of N pixels per side is obtained after a time equal to $N^2 \times t_{dwell}$ (green area in Figure S4a). The laser moves then back to the first pixel at the end of the image scanning. This return is done with a controlled time equal to $N \times t_{dwell}$. This time, represented in red in Figure S4a, is a dead time that slightly slows down the frame rate of the video finally obtained but this time is controlled and precisely known whatever the size of the image.

A complete sequence acquisition of 4 successive images is shown in Figure S4b. The time between each image is Δt. During this time, the particle has moved. With traditional particle tracking methods, the sampling time is fixed and the coordinates (X,Y) of the particle in the two successive images change (Figure S4c).

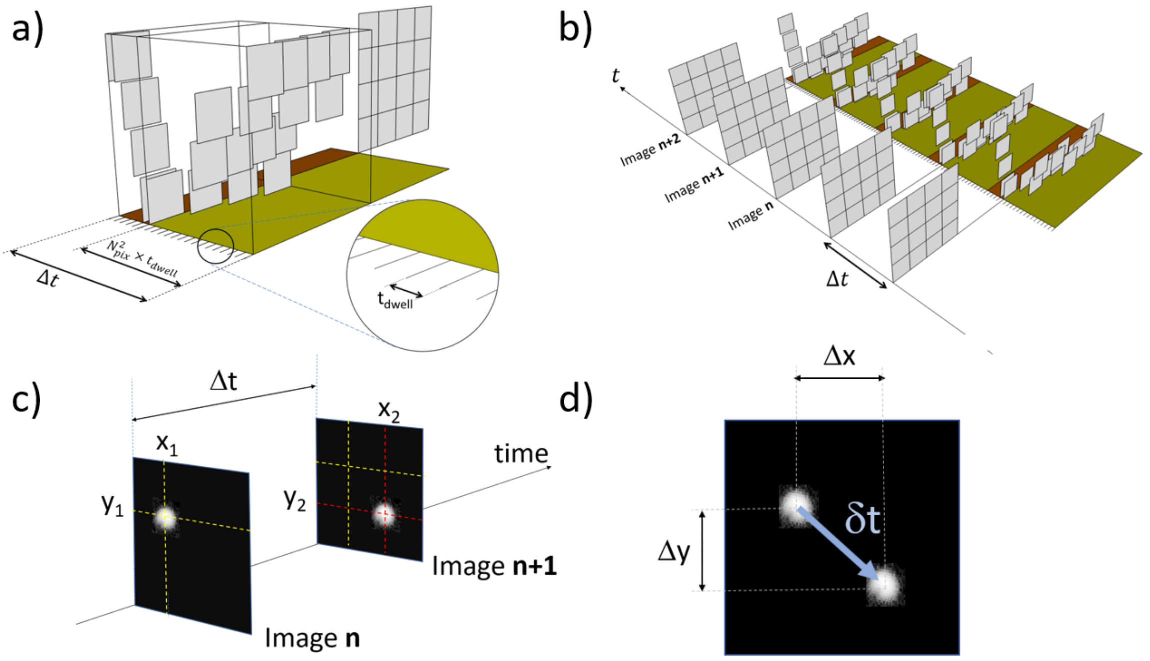

Figure S4. (**a**) timeline of one image acquisition, (**b**) timeline for acquisition of 4 successive images, (**c**) displacement of a particle between two successive images, (**d**) lag time between two images.

Here, we access the displacement of the particle between images but also to the variable lag time δt between two acquisitions. This variable, which can be positive or negative, is illustrated in Figure S4d.

4. Raw images

The scanning method used to build images limits the signal intensity received on each pixel. The raw images contain very few information because the photomultiplier only counts a few photons for a short dwell time (5μs). The images only use 2–4% of the grey scale dynamic range as represented with an enhanced intensity in Figure S5 in which the maximum raw intensity is 5 over 255.

This image shows the very small amount of information contained in an image of moving objects and the presence of noise coming from the excitation signal. The compromise between image resolution and frame rate mentioned in the main article is well represented here. The low resolution of the image makes difficult to accurately estimate the center of each bright spot. The dynamic range of this enhanced (Raw intensity multiplied by 55) image is shown with the histogram in Figure S6.

5. TrackMate process

An important step in the detection process is the choice of parameters in the TrackMate plugin. Below is described the procedure of detection from the raw images sequence until the generation of coordinates for every track.

1) In order to record several tracks, a sequence of 1000–2000 images is downloaded in the plugin TrackMate. For the first step of the detection, a type of detector must be chosen, in this study only the LoG (Laplacian of a Gaussian) detector is selected and makes sub-pixel localization possible.

2) To proceed to the detection, an expected blob diameter (purple circle) and a threshold are required to give the initial parameters for the LoG filter. An overestimated threshold will cause several gaps in the track when an underestimated threshold will result in false or erroneous positions detection. This parameter can be adjusted using a detection preview feature.

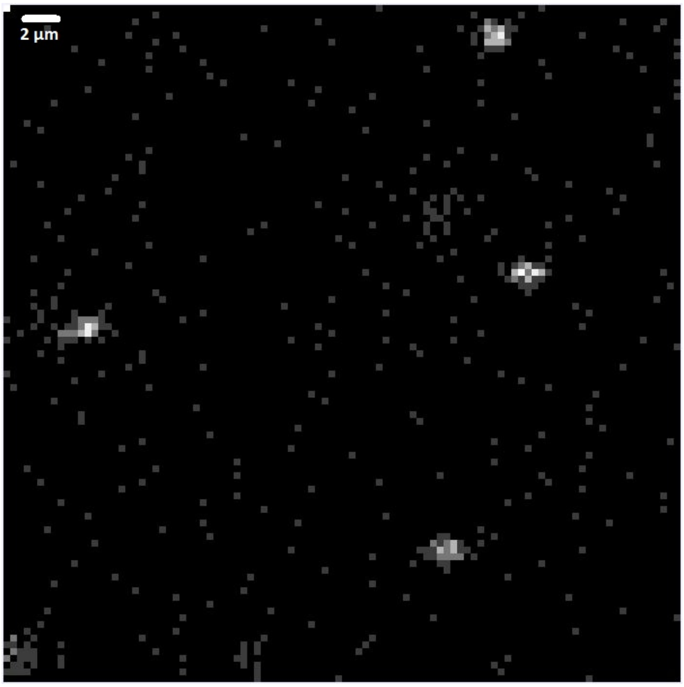

Figure S5. Raw image of latex beads of 500 µm of diameter in a droplet of water. Image 100x100 pixels, 2.53 pixels/µm, 5µs of dwell time and an objective x20 NA = 0.75. The image intensity have been multiplied by 30 to easily visualize the spots. The bright spots are the particles and the isolated grey dots are noise coming from the photomultiplier.

3) Then all the images of the sequence are searched for blob detection and if two spots are too close, the highest intensity is chosen.

4) When the blobs have been detected and localized, all the successive positions must be tested within a maximum link distance (i.e., search radius) represented by a faded orange circle centered on the blob. If the next image contains a similar blob in this area, then it is considered as the same particle. Every trajectory is build this way until the particle disappears. This maximum search radius is an important parameter depending on the velocity of the particle (i.e., the size of the particle). When particle size is known, it is easy to find this parameter using the maximum diffusion length equation (eq 20 on the main paper):

$$h_{max} = 3\sqrt{\pi D\, t} \tag{1}$$

5) The visualization of all the trajectories is important to detect unusual experimental conditions like important drift, aggregation of several particles, convection movement. But all the short trajectories (less than 100 positions) are not reliable for the estimation of the size.

6) In order to optimize the size estimation, only tracks with more than 100 positions are selected.

7) The last step of the process is to generate a text file with all the positions making up each tracks. This text file is then read by a program on Matlab for the size estimation.

6. Algorithm

Figure S8 and Figure S9 are simple description of the algorithm used to process the raw positions data for the method of constant time lag and variable time lag. The final result gives the estimation on the bead's radius. The drift represents a specific direction of displacement not coming from the brownian motion.

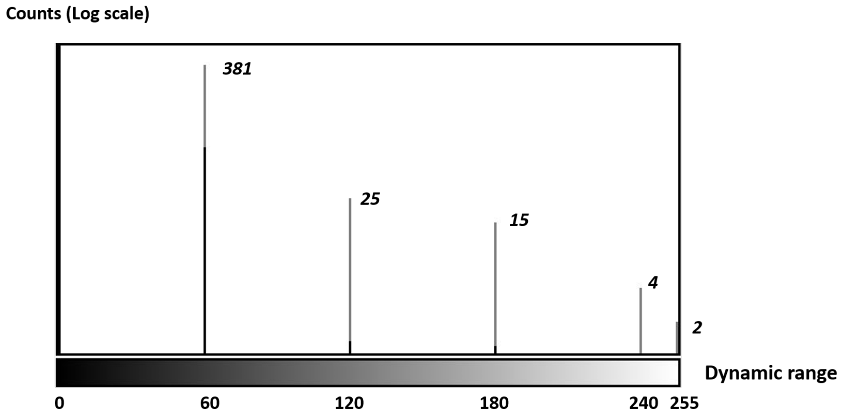

Figure S6. Histogram of the raw image Figure S5 represented with a Log scale for the counts. The raw intensity image were multiplied by 55 to be able to see all the bright spots and spread the diagram values.

7. Calculation of focal volume size via vectorial diffraction

7.1. Principle

As originally proposed by Richards and Wolf in 1959 [1], the excitation focal volume is computed by summing the interfering beams emanating from a single plane wave refracted at multiple angles by the microscope objective.

The interference, that is to say the sum, of all plane waves that converge to point x_2, y_2, z_2 of the focal plane can be written :

$$\mathbf{E}(x_2, y_2, z_2) = -\frac{ikC}{2pi} \iint_{\Omega} \mathbf{T}(s) \exp\left[ik(\Phi(s_x, s_y) + s_x x_2 + s_y y_2 + s_z z_2)\right] d\Omega \tag{2}$$

where :

- $\Phi(s_x, s_y)$ take into account eventual aberrations or the focalization through a dielectric medium like the glass coverslip [2].
- **s**, is the propagating vector of one plane wave. This plane waves are ordered around a cone and can be written in spherical coordinates as :

$$\mathbf{s} = (\sin\theta \cos\varphi, \sin\theta \sin\varphi, \cos\theta)$$

- Ω is the solid angle and

$$d\Omega = \frac{ds_x ds_y}{s_z} \sin\theta d\theta d\varphi$$

This can be sum up as :

$$\mathbf{E}(x_2, y_2, z_2) = -\frac{iC}{\lambda} \int_0^{\alpha} \int_0^{2\pi} \sin\theta A(\theta, \varphi) B(\theta, \varphi) \mathbf{P}(\theta, \varphi) \exp\left[ikn(z_2 \cos\theta + \sin\theta \cos\varphi x_2 + \sin\theta \sin\varphi y_2)\right] d\theta d\varphi \tag{3}$$

where :

- $A(\theta, \varphi)$ is the normalized amplitude of the optical beam before focalization. As a first approximation, it can be considered as uniform. More usually, it can be described by a gaussian :

$$A(\theta, \varphi) = e^{-2\frac{f^2 \tan^2\theta}{w^2}}$$

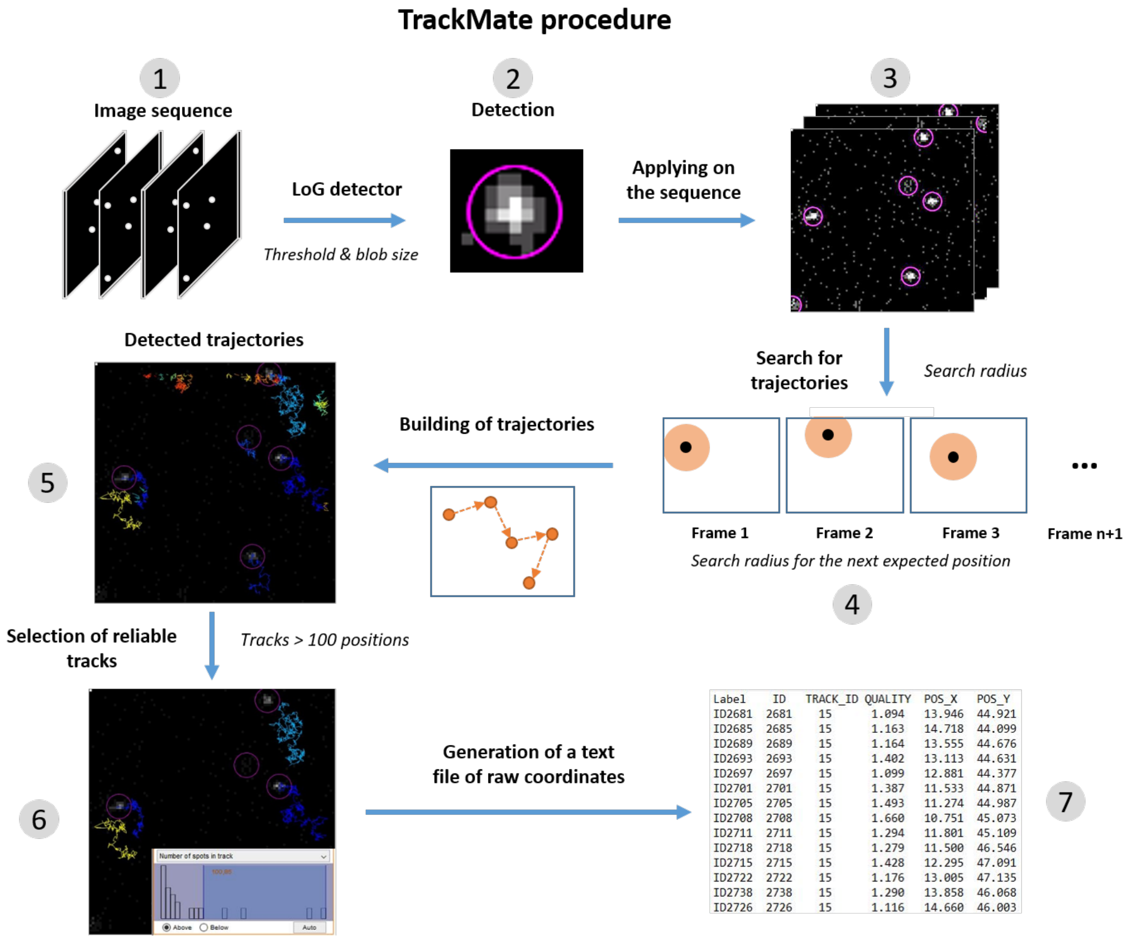

Label	ID	TRACK_ID	QUALITY	POS_X	POS_Y
ID2681	2681	15	1.094	13.946	44.921
ID2685	2685	15	1.163	14.718	44.099
ID2689	2689	15	1.164	13.555	44.676
ID2693	2693	15	1.402	13.113	44.631
ID2697	2697	15	1.099	12.881	44.377
ID2701	2701	15	1.387	11.533	44.871
ID2705	2705	15	1.493	11.274	44.987
ID2708	2708	15	1.660	10.751	45.073
ID2711	2711	15	1.294	11.801	45.109
ID2718	2718	15	1.279	11.500	46.546
ID2715	2715	15	1.428	12.295	47.091
ID2722	2722	15	1.176	13.005	47.135
ID2738	2738	15	1.290	13.858	46.068
ID2726	2726	15	1.116	14.660	46.003

Figure S7. Description of the whole procedure to generate the coordinates of the different particles along the tracks. The step 1 starts right after the record of a full sequence of images recorded by the Labview program. The procedure of TrackMate is over when the text file is generated and extracted.

where f is the focal distance of the objective lens and w is the waist of the laser beam.

- $B(\theta, \varphi)$ is a term that accounts for energy conservation for an aplanatic optical system : $B(\theta, \varphi) = \sqrt{\cos\theta}$ [3].
- $\mathbf{P}(\theta, \varphi)$ describe the polarization state inside the focal volume. It can be written as :

$$\mathbf{P}(\theta, \varphi) = T(\theta, \varphi)\mathbf{P_0}(\theta, \varphi)$$

with :

  – $T(\theta, \varphi)$ is a transformation matrix that permits to go from the plane right at the exit of the objective to the plane of the focal volume. $\mathbf{P_0}(\theta, \varphi)$ is the initial polarization state of the optical beam before focalization. The transformation matrix $T(\theta, \varphi)$ can be built from rotation matrices as :

$$T(\theta, \varphi) = R^{-1}CR$$

where :

$$R = \begin{bmatrix} \cos\varphi & \sin\varphi & 0 \\ -\sin\varphi & \cos\varphi & 0 \\ 0 & 0 & 1 \end{bmatrix} \quad , \quad C = \begin{bmatrix} \cos\theta & 0 & \sin\theta \\ 0 & 1 & 0 \\ -\sin\theta & 0 & \cos\theta \end{bmatrix}$$

R, C describe the rotation of the coordinate system around the optical axis and the change of polarization during the propagation through the lens [4].

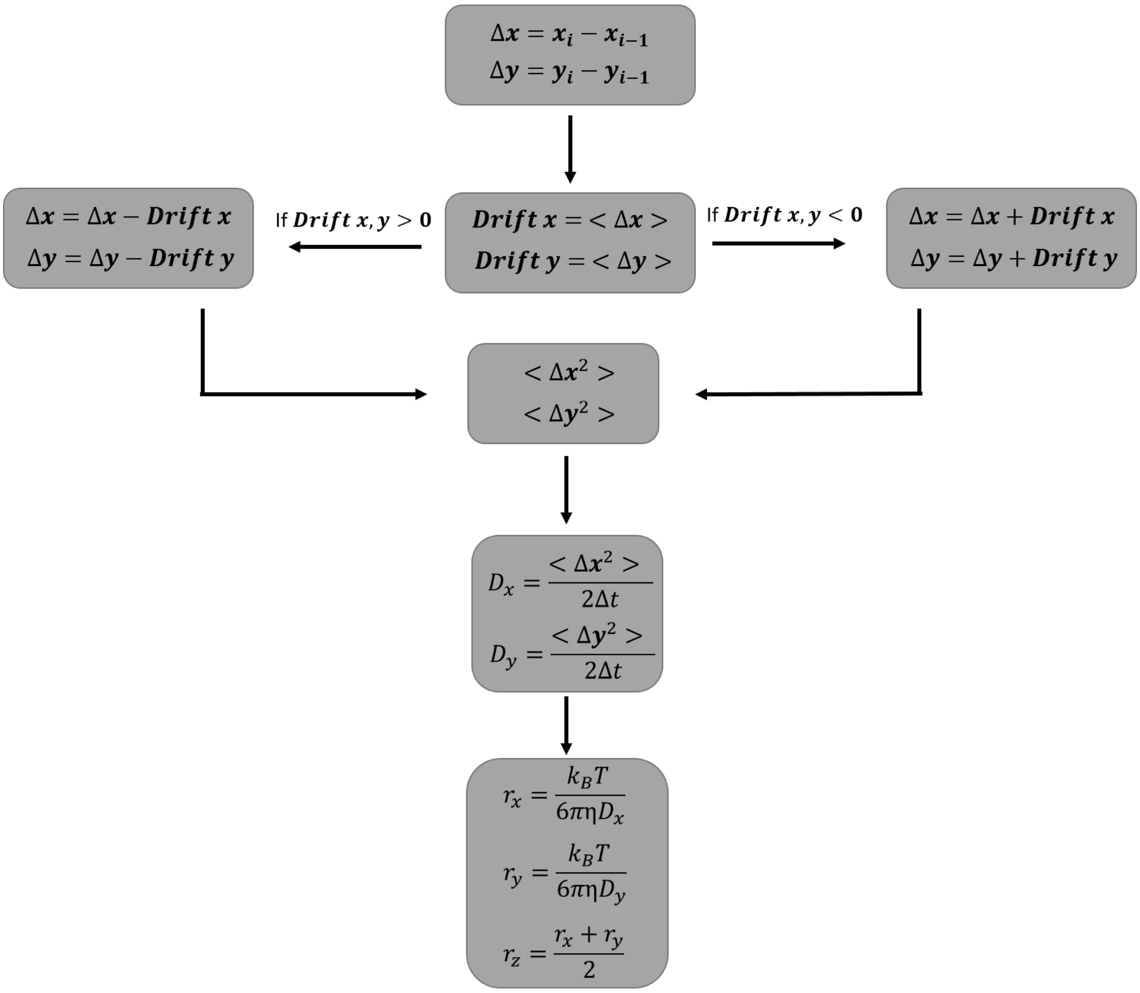

Figure S8. Flow chart of the algorithm using constant time lag to estimate the size of particles.

Further mathematical simplifications could be done assuming a gaussian profile for the laser beam $A(\theta, \varphi)$ and introducing Bessel functions. This would lead to faster calculation at the cost of generality [5].

Proceeding with the calculation of the rotation matrices, one can rewrite the polarization state $\mathbf{P}(\theta, \varphi)$ inside the focal volume as :

$$\mathbf{P}(\theta, \varphi) = \begin{bmatrix} 1 + (\cos\theta - 1)\cos^2\varphi & (\cos\theta - 1)\cos\varphi\sin\varphi & -\sin\theta\cos\varphi \\ (\cos\theta - 1)\cos\varphi\sin\varphi & 1 + (\cos\theta - 1)\sin^2\varphi & -\sin\theta\sin\varphi \\ \sin\theta\cos\varphi & -\sin\theta\sin\varphi & \cos\theta \end{bmatrix} \begin{bmatrix} p_x(\theta\varphi) \\ p_y(\theta\varphi) \\ p_z(\theta\varphi) \end{bmatrix}$$

7.2. Numerical procedure

Numerical calculation is done via discretization of the variations of θ and φ :

$$\mathbf{E}(x_2, y_2, z_2) = \sum_{m=1}^{N_\theta} \sum_{n=1}^{N_\varphi} \sin\theta_m A(\theta_m, \varphi_n) B(\theta_m, \varphi_n) \mathbf{P}(\theta_m, \varphi_n)$$
$$\exp\left[ikn(z_{2k}\cos\theta_m + \sin\theta_m\cos\varphi_n x_{2i} + \sin\theta_m\sin\varphi_n y_{2j})\right]\Delta\theta\Delta\varphi \quad (4)$$

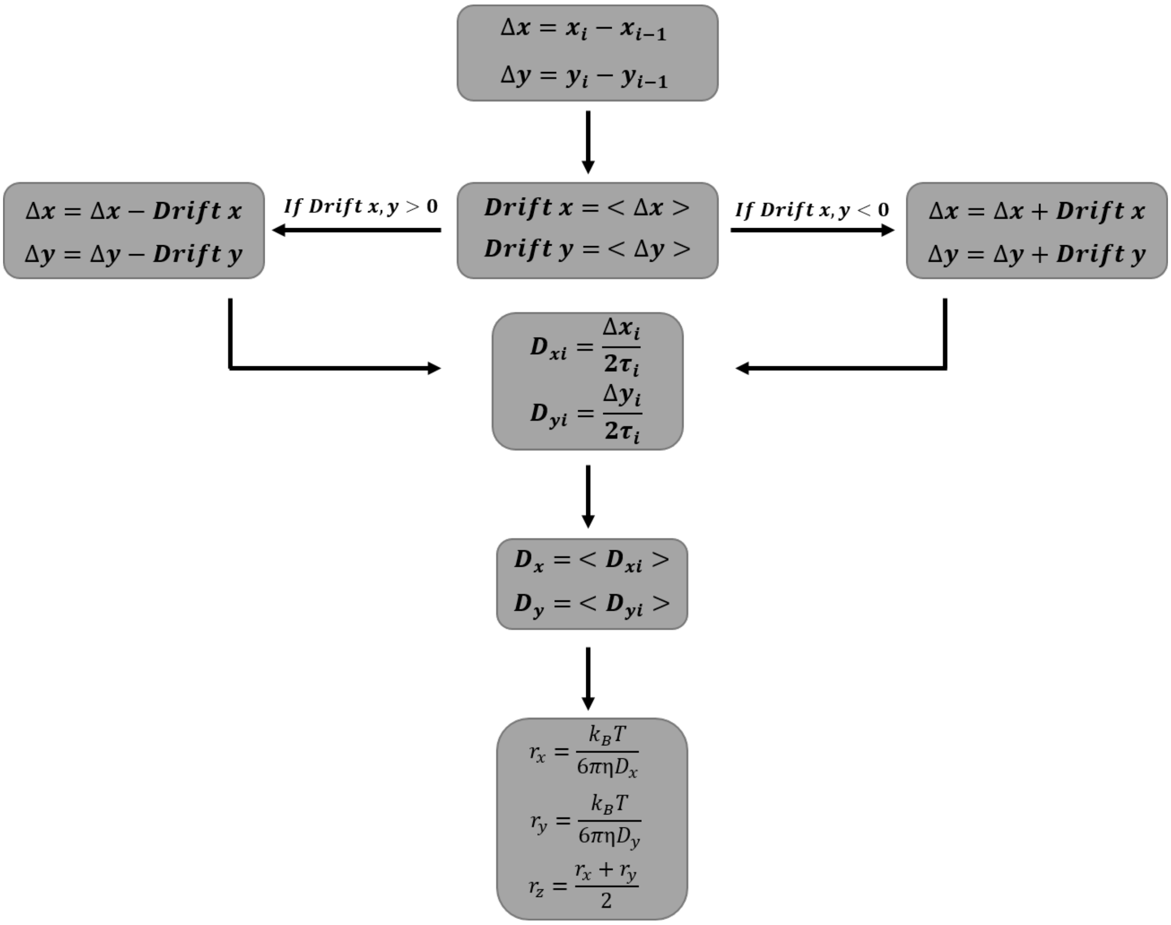

Figure S9. Flow chart of the algorithm using variable time lag to estimate the size of particles.

7.3. Unoptimized code

The following unoptimzed python code is loosely based on the matlab one that can be found in Qinggele Li's PhD thesis [6].

```
import matplotlib.pyplot as plt
import numpy as np

pi = np.pi

# basic optical parameters
NA = 0.75 #numerical aperture of objective lens
G = 20 # Grandissement
f = 180/G #focal length of the objective in mm
# (focal length of the tube lens is 180mm for Olympus)
n = 1 #refractive index of immersion medium
lambda_= 760.e-9 # wavelength of light
alpha = np.arcsin (NA / n)  # maximum open angle of OL
k = 2*pi*n/lambda_ # wavenumber

# image plane in Cartesian coordinates
L_focal = 20000.e-9; # observation scale
Nx = 3 # discretization of image plane
Ny = 3 # discretization of image plane
```

```
Nz = 200

# Waist of the laser beam in mm
w_centre = 3/2

x2 = np.linspace(-L_focal, L_focal , Nx)
y2 = np.linspace(-L_focal, L_focal , Ny)
z2 = np.linspace(-L_focal, L_focal , Nz)
# Return coordinate matrices from coordinate vectors.
XX, YY, ZZ = np.meshgrid (x2 , y2, z2)

E_x = np.zeros((Nx, Ny, Nz), dtype=np.complex64)
E_y = np.zeros((Nx, Ny, Nz), dtype=np.complex64)
E_z = np.zeros((Nx, Ny, Nz), dtype=np.complex64)

# normalization and steps of integral
N_theta = 500
N_phi = 50
delta_theta = alpha/N_theta
delta_phi = 2*pi/N_phi

theta = 0
phi = 0

# Linear Polarization
P = np.array([1,0,0])

for n_theta in range(N_theta):

    cosTheta = np.cos(theta)
    sinTheta = np.sin(theta)
    tanTheta = np.tan(theta)
    cosThetaSqrt = np.sqrt(cosTheta)

    # gaussian intensity profile of the laser beam.
    A = np.exp(-2*f**2*tanTheta**2/w_centre**2)

    phi = 0
    for n_phi in range(N_phi):
        cosPhi = np.cos(phi)
        sinPhi = np.sin(phi)

        a = 1 + (cosTheta-1) * (cosPhi)**2
        b = (cosTheta-1) * cosPhi * sinPhi
        c = -sinTheta * cosPhi
        d = 1 + (cosTheta-1)*(sinPhi)**2
        e = -sinTheta *sinPhi
        ff = cosTheta

        V = np.array([[a,b,c], [b, d, e], [-c, -e, ff]])
```

```
        # polarization in focal region
        PP = np.dot(V, P)

        common_term = = A * sinTheta*cosThetaSqrt* \
                    np.exp(1j * k * (ZZ*cosTheta + sinTheta*(XX*cosPhi + YY*sinPhi))) \
                    * delta_theta*delta_phi

        E_x += PP[0] * common_term
        E_y += PP[1] * common_term
        E_z += PP[2] * common_term

        phi += delta_phi

    theta += delta_theta

Ix = np.abs(E_x)**2
Iy = np.abs(E_y)**2
Iz = np.abs(E_z)**2

# Contour map in the focal plane
# plt.contourf(x2, y2, Ix[:,:,4], 30)
# plt.show()
# Iy2 = np.abs(Ey2)
# Iz2 = np.abs(Ez2)

I1 = Ix + Iy + Iz
z_profile_center = I1[int(Nx/2), int(Ny/2), :]
z_profile_center /= np.max(z_profile_center)
I1_biphoton = I1**2
z_profile_center_biphot = I1_biphoton[int(Nx/2), int(Ny/2), :]
z_profile_center /= np.max(z_profile_center)
plt.plot(z2*1e6, z_profile_center_biphot, label="Biphot")
plt.plot(z2*1e6, z_profile_center, label="Mono")
#plt.plot(z2*1e6, Ix[int(Nx/2), int(Ny/2), :]**2, label="Ix")
#plt.plot(z2*1e6, Iy[int(Nx/2), int(Ny/2), :]**2, label="Iy")
#plt.plot(z2*1e6, Iz[int(Nx/2), int(Ny/2), :]**2, label="Iz")
plt.legend()
plt.title("Squared laser intensity along the optical axis")
plt.xlabel("z / m")
plt.ylabel("Intensite")
plt.savefig("z_profile.png", dpi=300)
plt.show()
```

References

1. Richards, B.; Wolf, E.; Gabor, D. Electromagnetic diffraction in optical systems, II. Structure of the image field in an aplanatic system. *Proceedings of the Royal Society of London. Series A. Mathematical and Physical Sciences* **1959**, *253*, 358–379, [https://royalsocietypublishing.org/doi/pdf/10.1098/rspa.1959.0200]. doi:10.1098/rspa.1959.0200.

2. Kim, J.; Wang, Y.; Zhang, X. Calculation of vectorial diffraction in optical systems. *Journal of the Optical Society of America A* **2018**, *35*, 526. doi:10.1364/josaa.35.000526.
3. Youngworth, K.S.; Brown, T.G. Focusing of high numerical aperture cylindrical-vector beams. *Optics Express* **2000**, *7*, 77. doi:10.1364/oe.7.000077.
4. Helseth, L.E. Focusing of atoms with spatially localized light pulses. *Physical Review A - Atomic, Molecular, and Optical Physics* **2002**, *66*, 5. doi:10.1103/PhysRevA.66.053609.
5. Débarre, a.; Jaffiol, R.; Julien, C.; Tchénio, P.; Chaput, F.; Boilot, J.P.; Nutarelli, D.; Richard, A. Quantitative determination of the 3D dipole orientation of single molecules. *The European Physical Journal D - Atomic, Molecular and Optical Physics* **2004**, *28*, 67–77. doi:10.1140/epjd/e2003-00295-1.
6. Li, Q. Optimization of point spread function of a high numerical aperture objective lens : application to high resolution optical imaging and fabrication. Theses, École normale supérieure de Cachan - ENS Cachan, 2014.
